# Protocol for a matched-pair cluster control trial of ARCHES (Addressing Reproductive Coercion in Health Settings) among women and girls seeking contraceptive services from community-based clinics in Nairobi, Kenya

**DOI:** 10.1186/s12978-020-00916-9

**Published:** 2020-05-27

**Authors:** Jasmine Uysal, Nicole Carter, Nicole Johns, Sabrina Boyce, Wilson Liambila, Chi-Chi Undie, Esther Muketo, Jill Adhiambo, Kate Gray, Seri Wendoh, Jay G. Silverman

**Affiliations:** 1 Center on Gender Equity and Health, University of California San Diego, 9500 Gilman Drive, La Jolla, CA 92093 USA; 2 Population Council, Avenue 5, 3rd Floor, Rose Avenue, Nairobi, Kenya; 3 Family Health Options Kenya, Family Health Plaza, Mai Mahiu Rd, Nairobi, Kenya; 4 International Planned Parenthood Federation, 4 Newhams Row, London, SE1 3UZ UK

**Keywords:** Global health, Contraception, Gender-based violence, Reproductive coercion, Intimate partner violence, Sexual gender-based violence, Sub-Saharan Africa, Adaptation, Kenya, Protocol

## Abstract

**Background:**

Reproductive coercion (RC) and intimate partner violence (IPV) are prevalent forms of gender-based violence (GBV) associated with reduced female control over contraceptive use and subsequent unintended pregnancy. Although the World Health Organization has recommended the identification and support of GBV survivors within health services, few clinic-based models have been shown to reduce IPV or RC, particularly in low or middle-income countries (LMICs). To date, clinic-based GBV interventions have not been shown to reduce RC or unintended pregnancy in LMIC settings.

**Intervention:**

ARCHES (Addressing Reproductive Coercion in Health Settings) is a single-session, clinic-based model delivered within routine contraceptive counseling that has been demonstrated to reduce RC in the United States. ARCHES was adapted to the Kenyan context via a participatory process to reduce GBV and unintended pregnancy among women and girls seeking contraceptive services in this setting. Core elements of ARCHES include enhanced contraceptive counseling that addresses RC, opportunity for patient disclosure of RC and IPV (and subsequent warm referral to local services), and provision of a palm-sized educational booklet.

**Methods:**

A matched-pair cluster control trial is being conducted to assess whether the ARCHES intervention (treatment condition), as compared to standard-of-care contraceptive counseling (control condition), reduces RC and IPV, and improves contraceptive outcomes for woman and girls of reproductive age (15 to 49 years) seeking contraceptive services from community-based clinics in Nairobi, Kenya. All six clinics were assigned to intervention-control pairs based on similarities in patient volume and demographics, physical structure and neighborhood context. Survey data will be collected from patients immediately prior to their clinic visit (baseline, T1), immediately after their clinic visit (exit), and at 3- and 6-months post-visit (T2 and T3, respectively).

**Discussion:**

This study is the first to assess the efficacy of an adaptation of the ARCHES model to reduce GBV and improve reproductive health outside of the U.S., and one of only a small number of controlled trials to assess reductions in GBV associated with a clinic-based program in an LMIC context. Evidence from this trial will inform health system efforts to reduce GBV, and to enhance female contraceptive control and reproductive health in Kenya and globally.

**Trial registration:**

Registered May 23, 2018 - ClinicalTrials.gov, NCT03534401. Unique Protocol ID: 170084.

## Plain English summary

Gender-based violence, including reproductive coercion (RC; behaviors by male partners or others to interfere with women’s and girls’ contraceptive use and pregnancy decisions) and intimate partner violence (IPV; violence perpetrated by a current or former romantic partner), contribute to unintended pregnancy among women and girls globally. Few clinic-based model have been shown to reduce experiences of GBV, particularly in low or middle-income countries (LMICs). ARCHES (Addressing Reproductive Coercion in Health Settings) is a single-session intervention delivered within clinic-based contraceptive counseling session designed to address RC and IPV and has been demonstrated to reduce RC in the United States. ARCHES was adapted to the Kenyan context to reduce GBV and unintended pregnancy among women and girls seeking contraceptive services in this setting. This protocol describes the adaptation and evaluation of ARCHES underway in six community-based clinics (3 intervention, 3 control) in Nairobi, Kenya. This evaluation trial will assess whether ARCHES, as compared to routine contraceptive counseling, reduces RC and IPV, and improves contraceptive use and control among woman and girls of reproductive age (15 to 49 years). This study is the first to assess the efficacy of an adaptation of the ARCHES model outside of the U.S. Results from this study will be used to inform health system efforts to reduce GBV and improve the reproductive health and empowerment of women and girls in Kenya and globally.

## Background

Approximately 1 in 3 women worldwide will experience physical or sexual intimate partner violence (IPV) in their lifetime [1], an epidemic which contributes to female morbidity and mortality globally. Women and girls of reproductive age who seek contraceptives in health settings report higher rates of IPV than their same age peers [2–4], and those experiencing this form of gender-based violence (GBV) are significantly more likely to report unintended pregnancies and other poor sexual and reproductive health (SRH) outcomes [5–8]. Women and girls experiencing such abuse also report higher incidence of male partner opposition to their use of contraceptives [2, 9–11]. In 2018, the *Lancet Commission on Sexual and Reproductive Health and Rights* found that GBV was a significant contributor to global unmet need for contraception and unintended pregnancy, with the greatest proportion and relative impacts of GBV experienced by women and girls in low and middle-income countries (LMICs) [12].

Reproductive coercion (RC) is defined as controlling behaviors by male partners or family members to reduce women’s and girls’ reproductive autonomy [13] in the forms of limiting her access to or use of contraceptive methods, coercion to become pregnant against her will, and control of decisions to maintain or terminate a pregnancy [14]. A growing body of evidence demonstrates that RC is independently associated with unintended pregnancy, beyond the effects seen for IPV alone [2, 11, 15, 16], lending support to the hypothesis that this understudied form of GBV is a mechanistic link explaining, at least in part, the consistently-demonstrated associations between IPV and poor SRH outcomes [11]. While research describing the burden of RC in LMIC contexts is still at its inception, initial studies confirm the high prevalence of RC (as perpetrated by both male partners and in-laws) and its connection to experiences of unintended pregnancy [17] – an association which has been previously demonstrated in the United States [2, 8, 11].

In 2013, based on the growing understanding of the negative effects of GBV on the health of women and girls, and the need to address GBV as a human rights abuse, the World Health Organization (WHO) issued global guidelines recommending the identification and support of IPV survivors within health services [18]. This guidance was updated in 2019 to include the identification of RC [19]. Despite this global call-to-action, to date, only one clinic-based GBV prevention intervention has been shown via experimental or quasi-experimental design to reduce women’s experience of IPV in an LMIC context [20–22] and none have addressed RC or increased female contraceptive control. Although an important advance, this model was limited to women in the third trimester of pregnancy, and required [4] two-hour sessions with a counselor not providing routine care [22], making it challenging to integrate this intervention into existing routine health services.

ARCHES (Addressing Reproductive Coercion in Health Settings) is a brief, clinic-based intervention integrated within routine contraceptive counseling to address RC and IPV. It was developed and evaluated in the United States by a consortium of researchers from the University of Pittsburgh and the University of California San Diego, and practitioners from the U.S.-based NGO Futures without Violence. In two U.S.-based cluster randomized control trials (cRCTs) with over 4000 women and girls seeking care from reproductive health clinics, ARCHES was shown to reduce women’s and girls’ experiences of RC compared to standard-of-care contraceptive counseling [23, 24]. Importantly, women and girls receiving ARCHES also were more likely to report leaving a relationship that they considered unsafe or unhealthy, likely reducing their exposure to IPV, and to report increased knowledge and sharing of referral information for local IPV services [23, 24]. Based on this promising evidence, a consortium of researchers and practitioners led by the University of California San Diego (UCSD) and including the International Planned Parenthood Federation (IPPF), IPPF Africa Regional Office (ARO), Family Health Options Kenya (FHOK; NGO operating community-based health services), and Population Council in Kenya, set out to adapt and evaluate the ARCHES model in Nairobi, Kenya.

### Hypotheses

We hypothesize that women and girls age 15–49 years seeking contraceptives services from NGO-operated, community-based clinics in Nairobi, Kenya who receive the ARCHES intervention (treatment condition) will experience greater reductions in RC and IPV, and increases in uptake of modern contraceptive methods (primary outcomes), as compared to same age women and girls seeking these same services and receiving standard-of-care contraceptive services (control condition) at 3 and 6 months post-visit. We further hypothesize that the same women and girls receiving ARCHES will report decreased incident pregnancy (both overall and unintended) and attitudes accepting of RC and IPV and increased self-efficacy to use contraceptives (including in the face of RC), covert use of contraceptives (among those reporting RC), awareness and utilization of IPV services, and leaving a relationship because it felt unsafe or unhealthy as compared to those women and girls receiving standard-of-care services.

### Study overview and design

**Fig. 1:**
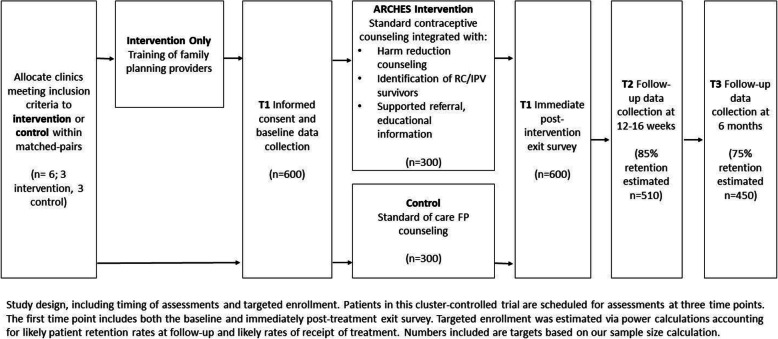
Study design

We will test our hypotheses using a matched-pair, cluster-controlled design with the two parallel treatment groups assigned in a 1:1 allocation ratio (ClinicalTrials.gov ID: NCT03534401). Eligible female patients seeking contraceptive care services from clinics allocated to the intervention or control condition will complete surveys at baseline prior to their clinical visit (T1), immediately after their visit before leaving the clinic (T2; i.e. exit survey), 12–16 weeks post-visit (T3; i.e. 3-month follow-up survey), and 23–26 weeks post-visit (T4; i.e. 6-month follow-up survey). We designed this superiority trial with sufficient power (*N* = 600) to assess our hypothesized short (3-month) and longer-term (6-month) outcomes. See Fig. 1. Study design and Table 1. Outcome measures.

**Table 1:** Outcome measures

Outcomes	Participant Survey measurement points				Analysis metric		Measures
	T1		T2	T3			
	*Baseline*	*Exit*	*3 month follow-up*	*6 month follow-up*	*Change from baseline to follow-up*	*Follow-up value only*	
*Primary outcomes for all participants*							
Reproductive Coercion in past 3 months	X		X	X	X		RC Scale (RCS; 9 items) [25] (binary)
Physical IPV and Sexual IPV in past 3 months	X		X	X	X		Conflict Tactics Scale-2 (CTS2; 7 items) [26] (binary) Sexual Experiences Survey (1 item) [27] (binary)
Uptake of female controlled contraceptive method		X				X	Self-report (1 item)
*Secondary outcomes for all participants*							
Incident and unintended pregnancy past 6 months			X	X		X	National Survey of Family Growth (NSFG; 1 item) [28, 29] (binary)
Self-efficacy to utilize contraceptives in the face of RC	X		X	X	X		Investigator-developed (4 items) [23, 24] (summary score)
Knowledge of IPV services					X		Investigator-developed (4 items) [23, 24] (summary score)
Reduced acceptability of RC and IPV	X		X	X	X		Investigator-developed (8 items) [23, 24] (summary score) Kenya Demographic and Health Survey (DHS) (7 items) [30] (summary score)
*Secondary outcomes for those reporting RC and/or IPV at baseline*							
Covert use of contraceptives in the face of RC in the past 3 months	X		X	X	X		WHO Multi-country Study on Women’s Health and Domestic Violence against Women (1 item) [10] (binary)
Utilization of IPV services among those reporting IPV in the past 3 months			X	X		X	Investigator-developed (8 items) [23, 24] (binary)
Leaving a relationship because it felt unsafe or unhealthy among those reporting RC or IPV in the past 3 months			X	X		X	Investigator-developed (1 item) [23, 24] (binary)

All measures were adapted for use in the current study via cognitive interviews with Kenyan contraceptive care patients and providers

## Methods

### Kenyan country context

Similar to many other LMIC contexts, unintended pregnancy is high in Kenya, with 25.4% of births being mistimed and 10.3% unwanted [30], and the more than 2 in 5 women reporting IPV in the country are at 70% increased odds of unintended pregnancy as compared to their same age peers [31]. More than 1 in 3 (36%) women report currently using a modern form of contraception [31]. Although no previous quantitative studies of RC have been conducted in Kenya, our qualitative formative research among women and girls seeking contraceptive services suggested that RC and IPV are highly prevalent in this context, and that RC appears to pose a major barrier to successful contraceptive use [32].

### Setting

Participants for this study will be recruited from private, community-based health clinics in the greater Nairobi area, owned and operated by Family Health Options Kenya (FHOK). FHOK is a local non-governmental organization (NGO) that has provided sexual and reproductive health service in the country for the past 50 years [33]. These community-based clinics provide comprehensive healthcare including contraceptive counseling, and offer a broad range of contraceptive methods. All FHOK clinics adhere to the Kenyan Ministry of Health (MOH) contraceptive counseling guidelines [33]. All six FHOK clinics located in slum and non-slum areas within and around Nairobi were selected to participate in the adaptation and evaluation of ARCHES. As the capital of Kenya and one of the largest cities in Sub-Saharan Africa, Nairobi has multiple local GBV support services available for women and girls at low to no cost [34].

### Description of intervention

#### Adapting ARCHES to the Kenyan context

Adaptation of the U.S.-based ARCHES model took a participatory and stepwise approach; details of the U.S.-based ARCHES intervention and evaluation findings are reported elsewhere [23, 24, 35]. First, formative research was conducted to understand specific forms of RC experienced by Kenyan women and girls seeking contraceptive services, strategies they used to cope with RC, and how these experiences were or were not addressed in the context of the health system. Qualitative data were collected in April 2017 from four of FHOK’s community-based clinics in the Nairobi area; including focus group discussions and in-depth interviews with women and girls aged 15 to 49 years-old. Semi-structured interviews with contraceptive service providers and clinic managers were conducted to understand providers’ experiences with patients reporting RC and IPV, and to identify further areas for adaptation and integration. Detailed methods and findings for this qualitative study are reported elsewhere [32].

Based on the results from the formative research and existing contraceptive care practice, the original ARCHES model was revised, and new materials and protocols were developed from June to October 2017. The study team workshopped the materials and protocols in weekly meetings with a consortium of local and IPPF GBV specialists and FHOK health providers. Through this process, adapted materials and protocols were further modified to fit within existing provider practices and responsibilities, and utilized pre-existing materials and protocols whenever possible. Additionally, materials for patients were reviewed via cognitive interviewing with a small convenience sample of women and girls seeking contraceptive services from FHOK. Once materials were further revised based on patient responses, a workshop was held with four FHOK providers to maximize the feasibility and acceptability of refined protocols via simulated counseling role-plays.

The adapted ARCHES program was then piloted in two FHOK clinics in slum neighborhoods of Nairobi. Five providers from the two facilities received a 3-day training on the adapted ARCHES clinical protocols and materials. Providers subsequently administered ARCHES to women and girls seeking contraceptive services for a 3-month period. Patients receiving ARCHES completed pre- and immediately post-intervention surveys to assess fidelity and quality of the intervention, as well as their perceptions of relevance, acceptability, and potential efficacy of this approach. Semi-structured interviews were conducted with the five trained providers to understand barriers and facilitators to implementation, and their perspectives on the acceptability and utility of the intervention to improve the reproductive autonomy of women and girls. Findings from the pilot indicated the feasibility of implementation of the intervention, and high levels of acceptability among providers and patients, along with suggestions for minor refinements to further facilitate faithful implementation [36]. Based on these findings, intervention protocols and materials were finalized for study implementation. The final adaptation included printed materials to aid providers in their implementation of ARCHES clinical protocols, patient education materials, a waiting room poster, a palm-sized educational booklet, as well as facilitator and provider training manuals and a training slide deck.

#### ARCHES intervention for implementation in Kenya

**Fig. 2:**
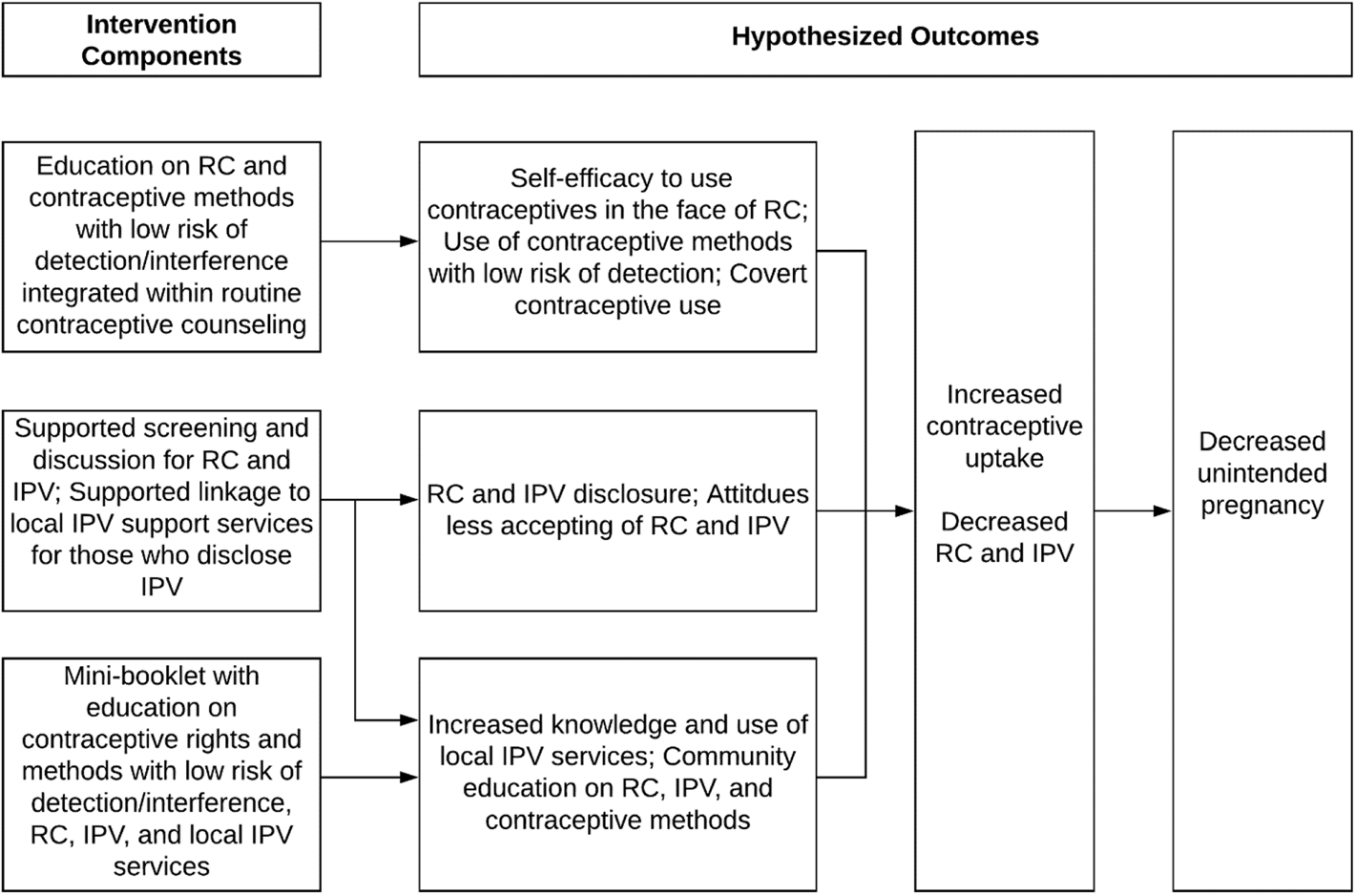
Conceptual model

The final intervention resulting from this adaptation process retains the three primary ARCHES strategies designed to educate and empower women and girls regarding contraceptive use in the face of male partner and family opposition, identify and address IPV and RC, and reduce unintended pregnancy (see Fig. 2. Conceptual model) which are universally delivered to all women and girls during private, contraceptive counseling.

In the ARCHES Kenya adaptation, all women and girls first receive comprehensive counseling and education on a broad range of contraceptive methods, and are asked about their pregnancy and contraception history and desires. Integrated within this otherwise standard contraceptive counseling is education on RC and contraceptive methods that may be used with a low risk of detection or interference from male partners or family members (i.e., contraceptive methods that may be used covertly, and strategies to reduce the likelihood of detection when using these methods) if they or someone that they know is facing RC. The specific strategies for reducing risk of detection included in ARCHES counseling are those that local women reported utilizing in the formative research [32]. Messages that destigmatize RC and affirm a woman’s right to use contraception, regardless of opposition, are also integrated.

The second ARCHES element involves providers offering an opportunity for a female patient to disclose and discuss their experiences of RC and IPV. Messages normalizing and destigmatizing RC (e.g., “Many of our patients have men or family members in their lives that make it difficult for them to use contraception.”) are delivered, followed by three standard screening questions regarding IPV (physical, sexual and emotional) and two standard screening questions regarding RC (pregnancy coercion and birth control sabotage). This element is designed to be a natural extension of the initial contraceptive counseling facilitated by the trust developed between the patient and provider during that initial discussion. ARCHES providers are trained to respond in a supportive and validating manner, and to never coerce or pressure a patient in any way to disclose either IPV or RC. This approach acknowledges that many women and girls do not disclose abuse to providers, or feel that is safe or appropriate to seek help from providers for GBV [37–39]. Women who do disclose IPV are offered to be connected by the provider via phone to a trained counselor from a local GBV survivor support agency at the close of their clinic visit (i.e. a “warm referral”); this approach has been shown to reduce barriers to referral uptake (trust of service providers, perception that services are not appropriate for their situation) [40, 41].

The final element of the model is offering all women and girls, regardless of disclosure, palm-sized educational booklets (available in English and Kiswahili) to take for themselves or to share with other women and girls in their communities. This booklet provides information about RC and IPV (with questions to reflect on personal GBV experiences), contraceptive methods that can be used with a low risk of detection, and contact information for local IPV support services. In U.S.-based efficacy trials, these educational booklets were highly effective tools for women to disseminate information and resources on GBV with other women in their families and communities [23, 24].

Due to the potential harm to patients that may result from discussion and disclosure regarding RC and IPV, providers are trained to always prioritize patient confidentiality, and to only administer ARCHES protocols when visual and auditory privacy can be ensured. If male partners or family members accompany a patient to the counseling room, providers must request to meet with the patient alone based on the premise of needing to ask potentially embarrassing questions. If the patient wishes for the accompanying male partner or family member to stay, or if they refuse to leave, providers are trained to only provide standard-of-care contraceptive counseling.

### Assignment of clinics to treatment conditions

As ARCHES facilitates changes in providers’ contraceptive service delivery to all female patients seen, individual random assignment of patients is not possible. Instead, assignment at the clinic level is required. All FHOK community-based clinics within and around the Nairobi area were enumerated (*N* = 6) by UCSD researchers and assigned to treatment or control condition in matched-pairs (*n* = 3 intervention, 3 control) by the UCSD and FHOK, with pairings determined based on those clinics most closely matched on patient volume, patient characteristics (i.e. wealth, age, services sought), and community type (slum vs non-slum). Random assignment within pairs was not acceptable to program implementers due to the reduced feasibility of providing regular support to ensure faithful, high-quality implementation to providers in distal clinics; i.e., those clinics closest to FHOK headquarters in southeast Nairobi were more likely to be assigned to the treatment condition. Participants self-assign to the treatment or control condition based on the clinic they visit for contraceptive services and enroll into the study by Population Council research assistants (RAs). No blinding of participants or providers will occur.

#### Training of intervention site providers

FHOK contraceptive counseling providers at clinics assigned to implement the intervention will be trained to deliver the adapted ARCHES model via a 3-day training (6 h of instruction per day). The ARCHES training utilizes Social Cognitive Theory, increasing providers’ behavioral capacity and self-efficacy to deliver ARCHES and services for contraceptive patients experiencing GBV through education, observational learning (peer-based), and practice. The training curriculum includes: [1] education on GBV (inclusive of RC and IPV), including patient narratives gathered during formative research [2]; values clarification exercises to clarify and challenge provider attitudes that pose barriers to women- and patient-centered care via group discussion and reflection [3]; instruction on ARCHES clinical protocols and local GBV services; and 4) demonstrations followed by group practice via role-plays and peer coaching. GBV education and values clarification are completed on day one, and clinical instruction and practice occur on days two and three. Providers trained during the pilot period are trained as facilitators in a 1-day training-of-trainers (TOT) to increase sustainability and scalability of the model in Kenya and encourage peer-support to improve adoption of and fidelity to the intervention.

Post-training, providers will receive clinic-based weekly technical assistance sessions for the first 3-months of implementation.

#### Assessing fidelity to the intervention

Research staff will compile implementation data summary reports based on weekly analysis of patients’ exit survey data to track intervention fidelity. Implementation data summary reports will include patients’ reported receipt of each intervention element. These results will be used to inform technical assistance sessions. Implementation data summary reports from control clinics will also be used to detect possible cross-site contamination.

#### Control condition

The three clinics assigned to the control condition will receive no additional training, thus, patients will receive standard contraceptive counseling based on their previous completion of training on contraceptive service delivery by the Kenyan MOH. If results of the ARCHES trial in Kenya prove the intervention efficacious, all control sites will receive ARCHES training and materials as part of FHOK’s plan to scale the intervention across its community-based clinics nationally.

### Outcome measures

All primary and secondary outcome measures are explained below and listed in Table 1.

### Primary outcomes

#### Reproductive coercion (RC)

Recent (prior 3 months) experience of RC will be assessed using a 9-item scale on specific RC behaviors including pregnancy coercion (e.g. “Has a male partner ever tried to force or pressure you to become pregnant when you did not want to be pregnant?”), and birth control sabotage (e.g. “Has a partner ever destroyed, hidden, or taken away your family planning method?”). RC is assessed primarily as a binary (yes/no) outcome based on a positive response to any RC experiences. Additionally, a summary score of the number of RC items experienced (Score 0–9) will be assessed in exploratory analyses. Items for this measure were adapted from those in the RC scale developed in the U.S. (Cronbach alpha = 0.74) [25] based on formative research and cognitive interviewing.

#### Physical and sexual intimate partner violence (IPV)

Recent (prior 3 months) experiences of physical IPV will be measured using an adapted version of the injury subscale of the Conflict Tactics Scale 2 (CTS-2) [26]. Physical IPV is assessed as a binary measure (yes/no) based on at least one positive response to a 6-item question series experiencing any of the included physically violent behaviors from an intimate partner (i.e. Has a male partner ever … pushed you, shaken you, or thrown something at you, slapped you, twisted your arm or pulled your hair, hit you with his fist or something that could hurt, kicked, dragged, or beaten you up, choked or burned you). Recent (prior 3 months) experiences of sexual IPV will be measured using a single item modified from the Sexual Experiences Short-Form Survey, “Has a male partner ever forced you to have sex or do something sexual when you didn’t want to?” [27].

#### Uptake of a modern contraceptive method

Report of receiving a modern contraceptive method during the clinic visit (binary, yes/no) will be assessed using a single item, “Did you receive a family planning method today from your provider?” with confirmation provided via a follow-up question regarding which method they received from a list of all available methods (i.e. IUD, implants, injection, contraceptive pills). The patch, vaginal ring and female condom are not included as they are not commonly utilized or available methods in this setting. Additionally, because the ARCHES intervention focuses on increasing uptake and understanding of female controlled contraceptive methods that are easy to use discretely to reduce RC, the male condom will be excluded from this measure.

### Secondary outcome measures

#### Incident pregnancy and unintended pregnancy

Incident pregnancy (prior 6 months; binary, yes/no) will be measured via one self-report item asking how many times the client had been pregnant in the past 6 months including stillbirths, miscarriages, and abortions. The single-item measure for 6-month unintended pregnancy was adapted from a 3-item assessment from the National Survey of Family Growth (NSFG) [28, 42]. The question is “At the time you became pregnant, did you want to become pregnant then, did you want to wait to become pregnant at a later date, or did you not want any more children?” Women who have never been pregnant will be considered to have not had an unintended pregnancy. Incident and unintended pregnancy within the past 6-months will be measured at follow-up visits only (T2 and T3). Unintended pregnancy (binary, yes/no) will be included as an exploratory outcome as the study will not be powered to detect a significant effect for this lower-prevalence outcome at alpha <.05.

#### Self-efficacy to use contraceptives, including in the face of RC

Self-efficacy to utilize contraceptives, including in the face of RC will be assessed using four items on participants’ confidence in their ability to successfully use a contraceptive method, including in the face of RC (e.g. “How confident are you that you could talk to your partner about your family planning use?”, “How confident are you in your ability to use family planning even if your partner tries to interfere?”). Participants are asked to rate their confidence regarding each statement on a 3-point Likert scale (Strongly agree, somewhat agree, disagree). The outcome will be modeled using a sum score (range 0 to 8). This scale, utilized in the U.S. ARCHES studies (Cronbach alpha = 0.72) [35], was modified for the Kenyan context based on formative research.

#### Acceptability of RC and IPV

Acceptability of RC will be assessed via an 8-item scale. Participants will be asked to endorse if, in their opinion, it is acceptable for husbands or male partners to enact different forms of RC (e.g. “force or pressure women to become pregnant or make it difficult for them to use family planning,” “throw away, destroy, hide, or otherwise interfere with a woman’s family planning method”) in a variety of situations (e.g. “if he wants more children than his partner”, “if she is experiencing side effects he does not like”). This Demographic and Health Survey (DHS) [30] wife-beating justification scale was modified to reflect acceptability of RC, and was piloted via cognitive interviewing. Acceptability of IPV will be assessed via 7 questions adapted from the DHS scale referenced above. Participants will be asked to endorse if, in their opinion, it is acceptable for husbands to beat their wife in a variety of situations (“she goes out without telling him,” “she argues with him,” “she burns the food”). Two additional situational question items will be added to the original scale that are relevant to RC (“she refuses to get pregnant,” “she uses family planning without his knowledge”). Participants are asked to endorse if they agree or disagree with the above statements. Question items will be added up for each scale (1 = agree; 0 = disagree) for a summary score where higher scores equate to attitudes more accepting of RC and IPV (maximum score 8 for RC; 7 for IPV).

#### Awareness of IPV services

To assess awareness of local services for women and girls experiencing IPV, participants will be presented a list of four local IPV support service agencies and asked whether they, “think a woman experiencing physical or sexual violence from her male partner could get help” at each one, with responses of yes, no, or don’t know. A summary score is calculated with one point per resource selected "yes" for a maximum score of 4. A similar list, utilized in the U.S. ARCHES studies (Cronbach alpha = 0.80) [35], was adapted to the Kenyan context based on the IPV services available in Nairobi.

#### Secondary outcome measures for those reporting IPV or RC at baseline

In addition to the outcomes above, three additional outcomes will be assessed among women and girls reporting ever experiencing IPV and/or RC at baseline, as this group is hypothesized to receive the greatest benefit from the ARCHES intervention.

#### Covert use of contraceptives among those reporting RC

Participants will be asked if they, “have ever used family planning without telling a male partner?” and a follow-up question establishes whether this covert use was within the past 3 or 6 months. Analyses of this outcome will be limited to those reporting ever having experienced RC at baseline.

#### Utilization of IPV services among those reporting IPV

Participants who report ever experiencing IPV at baseline will be asked whether they contacted or visited any local IPV support service agency in the past 3 and 6 months from the same list presented for awareness of IPV support services (see above). This will be modeled as a binary outcome, with participants who have called and/or visited any of the services being coded as having utilized IPV services.

#### Leaving a relationship because it felt unhealthy, unsafe or abusive among those reporting either RC or IPV

Participants, who report ever experiencing physical or sexual IPV or RC at baseline will be asked if, “in the last three months, have you left a relationship because it felt unhealthy, unsafe, or abusive?” This outcome will be assessed only at T2 and T3, and is based on a similar measure used in U.S.-based ARCHES trial [23, 24].

#### Demographics and other potential confounders

Demographics and potential confounders will include age, parity, food insecurity, marital status, highest level of education attended, and language of survey administration. Age will be assessed as a continuous measure. Other measures will be categorical in nature. Parity will be classified as either having no live births, having one live birth, or having multiple live births. Past month food insecurity will be assessed as a binary (yes/no) item based on the question, “In the last 30 days, did you or any members of your household go without eating the whole day because there was not enough food?” Women both formally married and those cohabitating with a male partner will be considered married based on common practice. Highest level of schooling attended will be classified as primary or lower, secondary, or post-secondary.

### Power calculations and sample size

Based on the pilot data from 143 female FHOK patients ages 15–49 years, prevalence of RC within the past 12 months was estimated at 17.1%. In a large U.S. cRCT, clients receiving the ARCHES intervention experienced a 0.4 reduction in odds (equivalent to an 8 percentage point reduction in intervention group reporting) of RC during the prior 3 months [23]. The present study is designed to achieve 80% power to detect a reduction of this magnitude (OR 0.4) related to past 3-month RC experience. Calculations assumed three control and three intervention clusters. Cluster size was assumed to differ in the range of 50–250 individuals per cluster, with a mean number per cluster of 100; the coefficient of variation for cluster sizes was thus set at 0.5. We anticipated low intra-class correlation (ICC) based on previous ARCHES trials (ICC < 0.01%) [23]. To be somewhat more conservative, we assumed an ICC of 0.1%. Under these parameters, the required average cluster size was 68, for a total of 204 control and 204 intervention participants (total required *N* = 408) retained at final follow-up. In previous ARCHES trials, retention was > 90% at 3-months follow-up; we assumed 85% retention at 3-months follow-up relative to baseline and 75% retention at 6-months follow-up relative to baseline, for an initial minimum N of 544. Our target final sample of 600 individuals exceeds the sample size required based on this conservative calculation.

### Participants – ethics, recruitment, eligibility, and informed consent

#### Ethics approval

This research protocol was initially approved by the Population Council institutional Review Board (IRB) on January 18, 2017 (Protocol Number 797), the Human Research Protections Program (HRPP) at the University of California San Diego on February 7, 2017 (Protocol Number 170084), and by the Kenyatta National Hospital-University of Nairobi Ethics & Research Committee (KNH-UON ERC) on March 2, 2017 for 1 year. Approval is sought from all ethics committees for amendments and annual renewals, which are currently approved through January 9, 2021. Research ethical and safety protocols for this study adhere to recommendations issued by the World Health Organization (WHO) for research on violence against women [43]. Ethics review by all three institutions determined that the proposed research poses no more than minimal risk to participants as the intervention provided is “reasonably commensurate with those inherent in their actual or expected situations” [44].

#### Recruitment and eligibility

Upon entering the clinic, female patients check-in for their appointment at the front-desk with the clinic receptionist who will offer the option to participate in a “women’s health study.” If interested in hearing more, the patient will be referred to a trained RA to complete recruitment. The RA will escorts the patient, alone without accompanying male partners or family members, to a location within the clinic that ensures visual and auditory privacy where they provide a brief description of the study and conduct eligibility screening. To be eligible for study enrollment, the patient must indicate that they are a) visiting the clinic “for family planning or are interested in receiving family planning” at their appointment that day, b) biologically female, c) age 15–49 years, d) not currently pregnant (self-report), e) not sterilized (self-report), f) have a male partner with whom they have had sex in the past 3 months, g) able to safely participate in a private interview without accompanying male partners or family members present, h) available for the 45 min necessary to conduct the interview, i) have no plans to move out of the area in the next 6 months, and j) have a mobile phone that can be safely used for re-contacting for follow-up surveys. As clients are recruited from general care, to be eligible for inclusion in follow-up surveys, participants must complete the immediate post-intervention (i.e. exit survey) and report having received contraceptive counseling on their exit survey.

If the patient declines to participate or is ineligible, the RA will walk them back to the waiting room and ensure that the patient did not lose their place in the queue for services. If eligible and interested, patients will receive a detailed explanation of the study, including estimated time required (30–45 min for baseline, 15 min for exit survey), compensation structure, that participation is completely voluntary and will not affect the care they receive, and that responses will be kept completely confidential. With permission, the RA will then administer informed consent and, after obtaining written informed consent, conduct the baseline survey in the language of the participants’ choosing (English or Kiswahili). After survey completion, RAs will remind participants of the 15-min exit survey to be completed before they leave the clinic and then walk participants back to the waiting room for their appointment ensuring they did not lose their place in the queue. After their visit with a provider, and before leaving the clinic, participants will meet with the RA for completion of the immediate post-visit exit survey. At the end of the exit survey, the RA will thank the participants for their time, schedule their 3-month follow-up survey, validate their contact information, and obtain an alternate contact in case they are unreachable.

#### Informed consent

Participants complete written informed consent to participate in this research. Consistent with Kenyan and U.S. law [44–46], local Kenyan and UCSD IRBs approved a waiver of parental consent for youth aged 15–17 years wishing to participate in the study after determining that the research poses no more than minimal risk to participants. This waiver protects adolescent girls seeking confidential reproductive health services at these clinics from a breach of confidentiality [45]. This waiver was also obtained to minimize any potential risk (e.g. retaliatory violence, expulsion from the home, or other harm) associated with breach of confidentiality to an abusive family members [47]. Completed and signed written informed consent forms will be stored in the Population Council’s Nairobi offices in a locked file cabinet until completion of data collection, after which time they will be destroyed.

### Data collection

#### Time points

Patients in both treatment and control groups will complete surveys at baseline (T1) prior to their clinical visit, immediately after their visit before exiting the clinic (exit survey), 12–16 weeks post-visit (3-month follow-up survey, T2), and 24–26 weeks post-visit (6-month follow-up survey, T3). RAs will be trained via a 5-day training, led by Population Council, that provides education on the ARCHES intervention, RC and IPV, responding to and referring participants that become upset during the interview, study protocols, and data collection forms. All surveys will be completed in-person via RAs reading all questions and responses aloud and recording responses using a tablet computer (Fig. 1. Study design; Table 1. Outcome measures).

#### Patient accrual and study flow

Six clinics, each treated as their own cluster, were matched-paired and allocated to a treatment group (3 intervention, 3 control). From each clinic, women will be approached for study recruitment; women will be excluded in the enrollment due to ineligibility or because they declined to participate, with the rest completing the baseline survey. See Fig. 1. Study design.

#### Re-contacting and retention

All patients that participated in the baseline survey, received contraceptive counseling, and completed the exit survey will be re-contacted to participate in a 3-month follow-up interview according to procedures designed to maximize participant retention. At the end of the 3-month follow-up survey, the RAs will schedule the 6-month interview and validate the patient’s contact information. All patients that participated in the baseline interview and met the eligibility criteria for inclusion in follow-up surveys will be re-contacted to participate in the 6-month follow-up interview, regardless of their participation at 3-months follow-up. Follow-up surveys will be conducted at the clinic where the participant was first enrolled into the study.

To maximize participant retention at follow-up surveys (T2 and T3), RAs will send text reminders approximately 4 weeks before their follow-up appointment to confirm contact and scheduling. Participants will also receive a text reminder the day before their appointment. If a participant is unreachable, RAs may attempt to reach them at their alternate contact (if provided). A maximum of five attempts will be made for each data collection point (T2 and T3). Participants that decline to participate will be asked why they are choosing to drop out of the study to inform loss to follow-up.

### Data analysis

We will adopt a difference-in-differences approach utilizing a multilevel, mixed-effects generalized linear regression, with logistic specifications for binary outcomes and linear specifications for continuous or scale outcomes, to evaluate associations between ARCHES intervention exposure and primary and secondary outcomes overtime. Longitudinal models will have fixed effects including time (baseline or follow-up), treatment arm, clinic, selected covariates, and a time and treatment interaction to indicate the treatment effect. These models will also take into account clusters (i.e. clinics) and clustering within clinics using nested random effects specifications to account for repeated measurements over time of individuals nested within clusters. The comparisons of outcomes at a single time point (e.g. immediately post-intervention) will use a similar multilevel mixed-effects regression approach, with single-level random effect specifications only for clinic, and only a fixed treatment effect (rather than time by treatment comparison). Final analyses will adjust for potentially relevant covariates (age, parity, food insecurity within the past 30 days, marital status, highest level of education attended, language of survey administration), in addition to time and treatment condition. Descriptive and bivariate analyses will be used to assess frequencies of and differences in demographics by treatment and loss to follow-up. Any characteristics identified as significantly associated with treatment or loss to follow-up at the *p* < 0.20 threshold will be considered as potential covariates (fixed effects) in adjusted models assessing effects of the intervention. Backwards selection at *p* < 0.20 will be used to finalize inclusion of potential covariates in regression models. Collinearity of covariates will be assessed, and highly collinear covariates may be removed from final models.

The primary analyses will use an intent-to-treat approach and analyze all subjects according to treatment group. Exploratory analyses will also be run stratified by age group (adolescent girls and young women age 16 to 24; adult women age 25 to 49). Missing data on demographics and birth history may be computed where alternate data is available (e.g. calculating age from date of birth if age is not provided); however, no missing data will be imputed. All scales to be used as outcomes will be assessed for internal reliability at baseline using exploratory factor analysis and Cronbach’s alpha. If scales are identified as multifactorial or not internally reliable, they may be modified to more accurately reflect the intended construct and/or be presented as individual item outcomes. An α level of 0.05 (i.e. 95% confidence intervals exclusive of one) will be considered the threshold for statistical significance in all analyses. Analyses will be conducted using STATA version 14.2® (StataCorp LLC, 2015) [48] and SAS version 9.4® (SAS Institute Inc., 2018) [49].

### Dissemination of results

Results about ARCHES efficacy to improve reproductive health outcomes and reduce RC and IPV among women and girls seeking services from community-based clinics in Nairobi, Kenya will be shared with the Kenya MOH, regional stakeholders, and IPPF member associations to inform scaling and replication efforts via presentations and reports. Results will also be shared with these and other research stakeholders at scientific conferences and via peer-reviewed publications in scientific journals. Publications will be open-access and available immediately after publication under the Bill & Melinda Gates Foundation Open Access Policy [50]. De-identified individual participant-level data (IPD) used for published analyses will be made immediately publically available via storage in an open-access data repository. Investigators may request access to the full data set. Authorship will be determined for publication based on the standards presented in the International Committee of Medical Journal Authorship [51].

### Data safety and monitoring plan

Precautions will be taken to ensure the safety of confidential data. During data collection, each patient will be assigned a unique participant identifier. Identifying information, including names and contact information of participants, will be used solely for the purposes of re-contacting participants for follow-up data collection. These data are stored in a secure file location separate from all survey data at the Population Council’s Nairobi offices, and are only accessible to Population Council investigators and study staff under their supervision. All identifying information will be destroyed once 6-month follow-up data collection is complete. Survey data will be uploaded from RA tablets daily via a secure internet connection and stored on a secure, encrypted server at Population Council, which is backed up nightly. De-identified data will be uploaded weekly to a password protected online server from which researchers at UCSD will download and store data to encrypted UCSD servers. These de-identified data will only be accessible to the PI and the trained research staff under his supervision. No survey data provided to UCSD will include any personal identifiers. Further, data shared with program partners (FHOK, IPPF) will include no identifiers to eliminate risk for loss of confidentiality. All data analyses will occur at UCSD; electronic files will be kept on a shared project drive within UCSD’s protected network. Data files on the online server and at UCSD are backed up every night to minimize the likelihood of lost files. Data files will be cleaned by UCSD analysts as data is uploaded weekly to remove duplicate observations and check data quality before analysis.

Data monitoring will proceed under direction of the PI in coordination with Co-Investigators at Population Council. If any privacy or data security arrangement is violated, the individual making the discovery will immediately notify senior research staff and the PI. UCSD, Population Council, and FHOK will take immediate action to identify the breach, remedy it, and terminate employment of anyone directly causing it. There are some circumstances in which confidentiality may be breached by the researchers. If the participant directly informs research staff of his/her intentions of homicide or suicide, the researcher will immediately contact authorities (police or mental health) to address the issue. This will be disclosed to participants during informed consent. Adverse events discovered by research staff will be reported to the PI who will then report the incident to the appropriate ethical review boards. Preliminary analyses, conducted monthly, will determine if the trial poses any threat or harm to participants. If these preliminary analyses reveal receipt of ARCHES is resulting in significantly worse reproductive health outcomes than standard-of-care treatment or increased violence the trial will be immediately suspended by the PI. In the U.S.-based ARCHES trials with over 4000 women and girls, no adverse events were reported to investigators.

## Discussion

This evaluation study protocol will guide assessment of the efficacy of the ARCHES intervention, as adapted and implemented in Kenya, to reduce IPV and RC and improve reproductive health outcomes among women and girls seeking contraceptive services at NGO-run clinics in Nairobi, Kenya. GBV, including RC and IPV, is a serious threat to the reproductive health and safety of women and girls globally and in Kenya. The U.S. ARCHES model has been found to reduce RC and IPV in two RCTs. This is the first study of the efficacy of ARCHES in an LMIC context. Evidence from this trial will inform efforts to address GBV and advance the reproductive health of women and girls in Kenya and other LMICs who experience a disproportionate burden of GBV.

## Data Availability

Not applicable.

## Abbreviations

ARCHES: Addressing Reproductive Coercion in Health Settings
UCSD: University of California San Diego
RAs: research assistants
RC: reproductive coercion
IPV: intimate partner violence
LMICs: low and middle income countries
FHOK: Family Health Options Kenya
IPPF: International Planned Parenthood Federation
GEH: Center on Gender Equity and Health
ARO: Africa Regional Office
MA: Member association
SRH: Sexual and reproductive health
NGO: Non-governmental organization
FWV: Futures without Violence
LARC: Long-acting reversible contraceptive
CPR: Contraceptive prevalence rate
MOH: Ministry of Health
IDI: In-depth interview
CCU: Covert Contraceptive Use
NSFG: National Survey of Family Growth
GBVRC: Gender Based Violence Resource Center
GVRC: Gender Violence Recovery Center
MSF: Médecins Sans Frontières
HRPP: Human Research Protections Program
cRCT: Cluster randomized controlled trial
IRB: Institutional review board
WHO: World Health Organization
IPD: Individual participant-level data
